# Supplementary material for: Patterns of Intron Gain and Loss in Fungi
Source: PLoS Biol. 2004 Nov 30;2(12):e422. doi: 10.1371/journal.pbio.0020422 (PMC532390; doi:10.1371/journal.pbio.0020422)
Supplement: Table S1 — Also available at http://genes.mit.edu/NielsenEtAl/. (4.3 MB ZIP). [file pbio.0020422.st001.zip › NielsenEtAl/html/535.html]

AN7572.1.NCU07378.1.MG00345.1.FG01312.1


```
 CLUSTAL W (1.82) Multiple Sequence Alignments - Introns Inserted


Sequence 1: NCU07378.1	1943 aa
Sequence 2: MG00345.1	1952 aa
Sequence 3: FG01312.1	1921 aa
Sequence 4: AN7572.1	2104 aa
Alignment Length: 2232 aa
Number Identitical Residues: 841 aa
Alignment Score (without introns) 40182


MG00345.1 	MDLPPASPAPALAEPTDSTPRAPSLGVQNGVPKTPNSLAPPAVSSLRADSAGMMRRSL--
NCU07378.1	---------------MEEQSKAPSL------------LAPPAVSSLRT---GKMERSV--
FG01312.1 	-------------MPMDSPSGAT--------------LAPPAVTSLRAQQTTAMERSL--
AN7572.1  	-------------MAGNDAENVQY-------------LTPPAVNALRQEARSVDPKNLPT
          	              . :.   .               *:****.:** .      :.:.:

MG00345.1 	----SEDIREEREELREAAEQTLNVIMDLNLDGRIRWVSPSWVDVIGTLPESVQGVAISD
NCU07378.1	----SENIREEREDLREAAEQTLNVIMDLNLDGTIRWVSPSWVDVIGTQPEQVTGTAITD
FG01312.1 	----SQDIREEREELREAAEQTLNTIVDLNFDGTIKWVSPSWTDVVGTQFETVNGAHISE
AN7572.1  	RTLSSDTMREERENLREAAEQTLNVIVDLDLEGRVKWVSPSWKQVVGTDLDTIERRLISD
          	 : :*: :*****:**********.*:**:::* ::****** :*:**  : :    *::

MG00345.1 	LIVSDNKTIFAEIVESMQKDDSKSQFIRFVVNLG--------PLSRLASLDAAMEGDAQP
NCU07378.1	LIVSDNKSIFADVIESMQRDDSKSKFIRFAVKVG--------PLSRLLPLEQTEEP----
FG01312.1 	LIVSDNKNVFTEVVESMKKDDSRSHRVRFTVQLG--------PLSKLLPIDELEHDPEDQ
AN7572.1  	VLVD-NKSAFLDAIESLKEDDSRSRFIRVSVQLGPDSVFKYAPAPRPLKREQTDGADSAA
          	::*. **. * : :**::.***:*: :*. *::*..:  . :* .:    :         

MG00345.1 	ER----------ETLELEAQGIMVYDTSSGGESH0TMWMVRPSIGPREIKIDLPDVIVES
NCU07378.1	------------TIIDVEAQGIMVYDGASGKDSH0TMWMIRPWVAPREIKIDLPAFIVES
FG01312.1 	PF----------HVVDLEAQGIMVYDSVSGGESH0TMWMIRPWVAPREIKIDLPNVIVDS
AN7572.1  	EEPSFEMEEDGPAILNLEGQGIMVYDRTAEGVAH0TMWMLRPYSKPREVTIDLPSSLVES
          	  .: . .....  :::*.*******  :   :* ****:**   ***:.****  :*:*

MG00345.1 	LGSGAEVLASYLTQLAETGLDDPAN--HAPPLPVLCRICERQIPPWWFEKHTDFCLQEHR
NCU07378.1	LGSGAEVLASYLTQLAESGLDDPNG--HPPPAPVLCRICERQIPPWWFEKHTDLCLQEHR
FG01312.1 	LGSGAEVLASYLTQLAESGLDDPEQ--LEPPPPVLCRICERQIPRWWFEKHTDLCLQEHR
AN7572.1  	LGVGAEVLANYLTVLAEAAANEPDQSKHPAPEPVLCRICERQITPWWFEKHSDLCLQEHQ
          	** ******.*** ***:. ::*  :.  .* ***********. ******:*:*****:

MG00345.1 	AEMDVQMAQESLTEHRHAIVKVLDALEVRKSRSLS---GDQSPLPLAEYKGFPIGPPPST
NCU07378.1	AEMDVQMTQESLIDHRHAIVKVLDALEARHSRTLI---GDPANLPIAEYKGMPIGPPTST
FG01312.1 	AEMDVQMAQENLAEHRHSIVKVLDALEARKSRSLA---GDQTPVPVAEYKGLPIGPPPST
AN7572.1  	AEMDVQIAQENLNEHRHAIVRVLDALEARQGRPISSRDGSSPHLPQPEYKGLPIGPSPVA
          	******::**.* :***:**:******.*:.*.: : .*. . :* .****:****.. :

MG00345.1 	TS----SSGASSPAPSNDRSSRERP----GHVRGRSFAVRRPQARIVELILDLCDTALEI
NCU07378.1	TS----SPGTSSPS-SNSGRSRDRSS-GFGHSRARSFAIRRPQARIVELLLDLCDTAIEI
FG01312.1 	QS----SPGSSLPR------SRERSG-GFGHTRARSFAIRRPQARIVELLMDLCDTAIEV
AN7572.1  	SAPSSGSTSTTSSAPATPPRSRDQSVSGSGHIRARSFAVRRPLARVVELILDLCDTALEI
          	 :.::.*..:: . .:.   **::. :. ** *.****:*** **:***::******:*:

MG00345.1 	SPPAIKEASS-TNPGEFRTQSPQSESRISQVMQWQSPSTNTLEQEQGLAMLCADTENVVK
NCU07378.1	STPAIKETT--QNPAEFRTQSPQSEGRISQVMQWETPGTNTLEQEQGLALLCTDTEAVAK
FG01312.1 	STPAIKESTTQQNNGEFRTQSPQSESRISQVLQWQSPSTNTLEQEQGLALLCADTEKVAR
AN7572.1  	NMPVLRESRG-DNGDEFRTMSPQSESRISQVLQWHSPSSNTLEQEQGLAALCTDTEQVAR
          	. *.::*:    *  **** *****.*****:**.:*.:********** **:*** *.:

MG00345.1 	AKVEAVLRHRRIIEYAERIRIEFAVTVEACIEEAMRKAAKIAAGRLSDSTED--------
NCU07378.1	AKVEAVFRHRRILEYAERIRIEFAIVVQECIDEAMRRAAKIAAGRLSDSTEE--------
FG01312.1 	TKVDAVFRHRKIIEYAERIRIELACLVQDCIDEALRKAARIANGELTDSTEEGRLHSDYE
AN7572.1  	SKVDAVIRHRRIVEYAERIRIEYTVLVEECITAALRKAERIAAGQLSDSSSS--------
          	:**:**:***:*:********* :  *: **  *:*:* :** *.*:**:..        

MG00345.1 	-----------EEEMCQAPAPDEIIETVGVD--------AATTSLALALQNAN---ISDD
NCU07378.1	-----------EEEEDFEQTEDEEATSAQEEG-IFPGSFDAPSTLALALEKAH---ISGE
FG01312.1 	HCDSCDEAVEADEDIPAEPLAPEPQQAEQRDSESLAGPLNSPSVLATALRQMS---LSEN
AN7572.1  	------EDETPSDETTSDPSASSASILVQNAG-HEPELPPPVSALTMSMRNATDRFQSGH
          	      .   ..::        .        .   .    . : *: ::.:  .   * .

MG00345.1 	PDRRRMSAAITSTRSSSPRECPTPRSHRGTLSISG-HTRESRRGSLLFESDPGDSDG-SM
NCU07378.1	RPSRRLSISIKSTRSSSPKECPTPRSHRGTLGNLA-SATQSRRESMLLESDAGDSDGGSI
FG01312.1 	RDRSRPTSFVASTRSSSPKECPTPRSHFGGQSNLGFQARDSRRDSIYMEGDFLDSEG-SF
AN7572.1  	SSEGKSSSAAVSTGPSSPMECPTPRSHKSVANLLG-TSQPSRRGLSLADIDSGDQSDSSI
          	    : :    ** .*** ******** .  .  .  :  ***     : *  *....*:

MG00345.1 	RSSSVTSRQPPRTESPISEFGDLRKQASSRQHHRRSLILPGAVS-PRRQESPSRGTQPSS
NCU07378.1	RSSSVASRMPTRTESPLSEFSDLRRHASSRQRNRRSLILPGTIS-PRRQESPSRNGPPSS
FG01312.1 	RSSSVTSRNAPRTESPISEFGDLRRAASTRRRHRRSLILPGTSS-PHRQESPSRPGQPSS
AN7572.1  	VSSSITG--AMRTDSPSSDR--------SLERKRRSLVLPGLSSSPRRQHSPARVSGPHS
          	 ***::.  . **:** *:         : .::****:***  *:*:**.**:*   * *

MG00345.1 	PLRIHKPRGLAFPSDALMSPESSPMLPGSEFTSPSLSHHHHHHRRQSSAAMSFANGEHGF
NCU07378.1	PLRLHKPRNLPFPSDSLVSPEASPMLPSSEFTSPTP-PSIHHHRRQSSAAVSASTGDLGF
FG01312.1 	PLRIK-ARGFPMSQEGVVSPEASPMLPGSDFSSPAA-HSHRHHRRQSSAAIS----DFVM
AN7572.1  	PLRMPKPR-LSTGAESLSSPIVS---PSSNLGELAL-HHYRHHRRQSSTTSS-----DLP
          	***: ..* :.   :.: **  *   *.*:: . :     :*******:: *        

MG00345.1 	RPPPSPRLSAANPPPLARAVPPSIKDFEVIKPISKGAFGSVYLSKKKSTGEYFAIKVLKK
NCU07378.1	KPPPSPRLSAVVGATQAKAVPPSIKDFEIIKPISKGAFGSVYLSKKKSTGEYFAIKVLKK
FG01312.1 	KPPPSPRLGANQAPPQQRPTQPSIKDFEIIKPISKGAFGSVYLSKKKSTGEYFAIKVLKK
AN7572.1  	RPPVSPHLTTAS-QPQPRPSPPSIKDFEIIKPISKGAFGSVYLSKKKTTGEYYAIKVLKK
          	:** **:* :    .  :.  *******:******************:****:*******

MG00345.1 	ADMVAKNQVTNVKAERAIMMWQGESEFVAKLYWTFSSKDYLYLVMEYLNGGDCASLIKIL
NCU07378.1	ADMVAKNQVTNVKAERAIMMWQGESDFVAKLYWTFSSKDYLYLVMEYLNGGDCASLIKVL
FG01312.1 	ADMVAKNQVGNVKAERAIMMWQGESDFVAKLYWTFSSKDYLYLVMEYLNGGDCASLIKVL
AN7572.1  	ADMVAKNQVTNVKAERAIMMWQGESDFVAKLYWTFSSKDYLYLVMEYLNGGDCASLVKIL
          	********* ***************:******************************:*:*

MG00345.1 	GGLPEDWVKKYLGEVILGVEHLHSRGIVHRDLKPDNLLIDQKGHLKLTDFGLSRMGLIGR
NCU07378.1	GGLPEDWVKKYLGEVVLGVEHLHSRGIVHRDLKPDNLLIDQKGHLKLTDFGLSRMGLVGR
FG01312.1 	GGLPEEWVKKYLGEVVLGVEHLHSRGIIHRDLKPDNLLIDQKGHLKLTDFGLSRLGLVGR
AN7572.1  	GGLPEDWTKKYIAEVVLGVEHLHSRGIVHRDLKPDNLLIDQKGHLKLTDFGLSRMGLVGR
          	*****:*.***:.**:***********:**************************:**:**

MG00345.1 	QKRALNSGTTDPAPDLLKQGPFARSASIASSRSTSLDLQAPSHSPGNTPHTNPSES--HL
NCU07378.1	QKRALNNG-ADAAPDLLKQGPFVRSSSIASSRSTSLDVHARNPSPLSTPQMTPSDYGAGL
FG01312.1 	QKRALNSEISDGTPDLLKQGPFARSASIASSRSASLDLHGHTQSPSQTPQMTPEVG--SL
AN7572.1  	QKRVLKSMNEQSG-DILKQGSFPRATSIASSRSASFDFQG-SGSPGSTPLITPDVAG-SM
          	***.*:.   :   *:****.* *::*******:*:*.:. . ** .**  .*.  .  :

MG00345.1 	AQPSYFSLGS--SKRDSRRFSG-HRSDSGGSEGLANMLSNFSLHDATSATVPPQAVQSPP
NCU07378.1	GQPSYFNLGV--LSQEPRRLCA-NRSDSGGSEALSHMLPNLSLNDTLS--VPPQAIQSP-
FG01312.1 	AQPSYFSLG-----QEPRRISGTHRSDSGGSETLARMVTNFSLNDAEVT-SQANSIKST-
AN7572.1  	PQPSYFNLNQSGLSRQPSRRASGYRSDSGGSENLNAIFRTLSLNEGETPGAMPIPPPPPS
          	 *****.*. :. .::. * ..  ******** *  :. .:**::   .   . .  ...

MG00345.1 	LADGSEGEGSGSPDPGSLHHTIGAHGSVSIEAGKGTPP--SNMPPPNWALFDPEDKNRRF
NCU07378.1	-GERSEADEGTTPDFVSLSHATTQS---NVDANRGTPPQQPHMPPPNWALFDPQDTNRRF
FG01312.1 	--LDEEAITQGSPD---LPMSVGSR--MSMDSHARNSLPLSNMMPPPMALFNPEDTNRRF
AN7572.1  	-DSAHEEEGQSEPAESPYLQPLQPT---HSNVSTGTPPQ-QTMMPPLMALFDPEDHNRRF
          	     *      *   .   .         :    ..     * **  ***:*:* ****

MG00345.1 	VGTPDYLAPETIKGDPQDETSDWWSVGCIMFEFLYGIPPFHAPEPELVFENILARRIQWP
NCU07378.1	VGTPDYLAPETIRGEPQDETSDWWSVGCIMYEFLYGIPPFHAPEAEQVFGNILARKLEFP
FG01312.1 	VGTPDYLAPETIKGDKQDETSDWWSVGCILFEFLYGIPPFHADEAEHVFENILARKIQWP
AN7572.1  	VGTPDYLAPETINGVGQDEMSDWWSLGCIMFEFLFGYPPFNAATPDEVFENILHRRINWP
          	************.*  *** *****:***::***:* ***:*  .: ** *** *::::*

MG00345.1 	EEMEDE-VSAEAKDILNKLLCMNPPERLGSNKDEKYPSGGEEIRSHPWFEGMNWDTLLQD
NCU07378.1	DDSDME-ISSEAKDLINKLLCSDPHQRLGANREDKFQSGGEEIRSHPWFEGVNWETLLQD
FG01312.1 	DENECEPISEAAKDLINKLLCMEPHQRLGSNQDDKFPSGGDEIRSHPWFQDVNWDTLLED
AN7572.1  	GEVEEL-ASPEAIDLMNKLMAINPRERIGANLEEKYPNGGAEIRSHPWFADIHWDTLLED
          	 : :    *  * *::***:. :* :*:*:* ::*: .** ******** .::*:***:*

MG00345.1 	EAQFVPQLENPEDTEYFDARGATLQSFAEEMEDQTSPPS-APASDYADRPHDALSRVRSQ
NCU07378.1	DAQFVPQPEHPEDTEYFDSRGAVLQSFAEEMEDQSSPPSSAPAPDYHNRPHDALAKVRSQ
FG01312.1 	EAQFVPQPENPEDTEYFDARGAVLQSFAEEMEDQASPQSSAAGSEYAERPHDALSRVRSQ
AN7572.1  	KAQFVPNLENPEDTEYFDARGATLQAFTEEDE--SSPQAPVTTGSYIDRPHDALYKVRSQ
          	.*****: *:********:***.**:*:** *  :** :...  .* :****** :****

MG00345.1 	FNS----MKRGLMPLHIPPHVRDLKSRRLSEPVAPDDFGNFSFKNLPVLDKANKDVIQKL
NCU07378.1	VNSNINTVKRGLMPLHIPPHVRDLKSRRLSEPVAADDFGNFSFKNLAVLEKANKDVIQRL
FG01312.1 	VNS----INRKLMPLHIPPHVRDLKSRRLSEPVAMDDFGSFSFKNLPVLEKANKDVIQKL
AN7572.1  	VNS----IKRPLMPLHIPPHVREPRSRRLSEPSLADDFGNFAFKNLPMLEKANKDVIQKL
          	.**    ::* ***********: :*******   ****.*:****.:*:********:*

MG00345.1 	RAEALAAQ--------NKPASISPGGLSVVTSPGGPSASMEGSPVLSMPVQRSLSNAKAS
NCU07378.1	RAEAAESQ--------TKP--ISPGALSSISSPG-TIGALEGSPVIANPVQRTLSAAKAS
FG01312.1 	RAEALAAQ--------SKGTSTSPGAVSSAVSPG---PSLEGSPVLSNPVSRTISNAKAT
AN7572.1  	RQEALQAQQRQSSNAGTQQQPQTQGQTQATGPTPPSGPSLDGSP-LPMSLQRTLSQSKGN
          	* **  :*. .::.:..:  . : *  .   ..  .  :::*** :. .:.*::* :*..

MG00345.1 	QRPQSPSGIGHN-ASPSRPSQPSSPLLVSFVANQGGEGRRKASSNSSSFSQPSSSS-LQP
NCU07378.1	QRPQSPSGIGHS-NSPNRASQPSSPLLVSFVAGQGAEGRRKASSNSSSLSHQSGS----F
FG01312.1 	NRAQSPSGFGHVSASPNRASQPSSPLLVSFVAGQGAEGRRKASSNSSSLSHQSTAATLQP
AN7572.1  	NRPASPSSLSQANSSPSRPSQPSSPLLVQFSTGHNHERRKTSGSSSTTASHQSSGS--FQ
          	:*. ***.:.: . **.*.*********.* :.:. * *:.:.*.*:: *: * .:    

MG00345.1 	GSFFEVPRVPPSLQKAAT--VAASPVKGR-PIPSPLPLSPQK------ILATPRQGSGSS
NCU07378.1	QSTVEGTRVPPTLQKAATTTTASSPSNGR-LVMPPLPLSPQRTVPAHTLTSSPRNTSGPA
FG01312.1 	SSAFEIPKVPPSLQKAATTVGASSPVQTRSAAPPPLSLSPQK------TITTPKQSSNSS
AN7572.1  	PASADQTRVANSNKFGSASSSPIKPNRVG-------THSPDK------SGPGPHRHGSAP
          	 :  : .:*. : : .:::  . .* .         . **::        . *:. ....

MG00345.1 	NTRSRSLTVGSQDG--SPVAS--DLLVHHRNRRSQVFDMSPSSSDNEGDKANALLRVQRR
NCU07378.1	TGRARSLTVGSQEGSGSPISA--DILAHHK-RRSQVFDMSPSSSDTEGDKANALLRVQRR
FG01312.1 	AGRSRSLTVGSQSQDGSPVAS--DIMHHHRNRRSQVFDMSPSSSDNEGEKHNALLRVQRR
AN7572.1  	STRTRSQTIGSQDGELSSSLARETYVPGHHKRRSQLFDVSPSSSDNEDPRAKALLKVQRR
          	  *:** *:***. . *.  : .  :  *:.****:**:******.*. : :***:****

MG00345.1 	RQSSRRMSQINLESTGPVFRPLDVLICEDHPVSRMIMEKLLEKLRCRTISASNGSEAVRY
NCU07378.1	RQSSRRMSQV---LDGPMFRPLDVLICEDHPVSRMVMEKLLEKLRCRTISASTGQEACRY
FG01312.1 	RQSSRRLSQIAF-DEGPTFRPLDVLICEDHPVSRMVMEKLLEKLRCRTISVPNGSEAVRY
AN7572.1  	RQSSRRLSQINF-ADGPFFRPLDILICEDHPISRLVMERLFEKLRCRTITVANGAEAVRY
          	******:**:     ** *****:*******:**::**:*:********:...* ** **

MG00345.1 	SMSDIKFDIIFMEFRLPQINGADVARMIRETKNSNSHTPIVAITAYLKELQAPHYFDSLI
NCU07378.1	ALSDIKFDIIFMEFKLPVINGSDVARMVRETKNKNHHTPIVAITAYLKELHAPHYFDSLI
FG01312.1 	SMSEIKFDVIFLEYQLPQINGADVARMIRETKNANSHTPIVAITAYLKELQAPHYFDSLI
AN7572.1  	ALSEVQFDIIMTEFKLPQVNGADVARMVRETRSANRHTPIIAVTSYLKDLPETHHFDSLI
          	::*:::**:*: *::** :**:*****:***:. * ****:*:*:***:*  .*:*****

MG00345.1 	DKPISSSKLTEVLRNLCQWKPPSSSQYSSLSLPLSHPAPSGLRQESSRLEDSP-TSGSSI
NCU07378.1	EKPISSSKLSEVLSTFCQWKPASPGQVQMLSLGLPPPVPSGLRQESLRLEDSP-VSAS--
FG01312.1 	EKPISSSKLTEVLQGLCRWRPESPG----HSSGMPRPVPLGIRKANSRLEDSP-TSGSSI
AN7572.1  	EKPPTLTKLTEVLCKFCQWKPPPKD--------YNPSMFAARQTPPTQSDHSPSSTVSSG
          	:** : :**:***  :*:*:* . .           .   . :    : :.**: : *: 

MG00345.1 	FAQRTGSSFRGSSREDSISSSLFGDSESVATDDIPVVISRTATGEWEE--GALGISQDEK
NCU07378.1	-GSSKYTVHSGGSVKEESTCSTFGDSESVATDDIPVVVSRKATGDWSE--GGLGISGSEE
FG01312.1 	FAGRLGAAMMS-SREDSITSSLFGDSESVTTEELPVVVSRKGTNEWDE--GGLNITENND
AN7572.1  	FALVPSSSYRGSSREESVGSSYFGDLDSIKAEEVPVIVSR-QTDDWSQGTGGLGISEDST
          	 .    :   ..* ::.  .* *** :*: ::::**::**  *.:*.:.:*.*.*: .. 

MG00345.1 	VLSS------------EPTKLSPMPIGAPPKTLS-------QQRSLEKLKAKREAIEKRR
NCU07378.1	VLVAGDD---GSKPPVHAQLLTQQSAPAQMEHLAGQTKGPVPQRSLDKLRARRESIEKRR
FG01312.1 	AHEP--------TKKAIPQLVAQQSAPAQMEHHI---------RVNDKVKGKREGSEQRT
AN7572.1  	SGQGSADSNRGAQNSTVPFPGLPHAAHSPPALSPSGMITPRKQRSSEAIRAKRESLERKR
          	    . .:. .:     .      .  :      .    .  .*  : ::.:**. *:: 

MG00345.1 	SDCSVDSADDEDEELGVSSLDSRSNSSSTSGSIHSGSSHSQSRQHFRSKSVLPSSKLGIE
NCU07378.1	LEGCIDSADDEDEELGAGQANS--------------GQEPHPAKHHRPKSTLPSSKLGIE
FG01312.1 	LEG-TESADDEDEELGGSREKHH-------------EHHGRNSQQMLSKASLPSSKLGIE
AN7572.1  	YEC-AESGDDEDEELG---------------------NLPSRTQSPRNRASRPGSKLGIE
          	 :   :*.********                           :    ::  *.******

MG00345.1 	MMRANSHDSVVFPPDATGSGDAELRE-----------------------SPLSQHDEEDE
NCU07378.1	MMRANSHDSMT-AADSEGASSATLGPPTA---------------QIVTPTTDVQMELPPL
FG01312.1 	MMRANSHDSLTVGSESTPEAVTQVVT-----------------------TPSKEMEAG--
AN7572.1  	MMRTNSRGSVISTSEDALKKDKEAGERSRSGGSDDADEHARDKIRSGRSSLESKFEDLNI
          	***:**:.*:   .:            : :..:..:.. : ..     .:   : :    

MG00345.1 	VGDTTVIHTPDQCGSSEDNATVVGVDETP--RPASD-------------SQFIADAGNDG
NCU07378.1	VVDTAAATMAPYTTVADDEAAAATVSTPPDLRPETE-------------GRKICHVEQTP
FG01312.1 	ILDGEIVAEPTASTPPDSEMKVEDI------TPKTD-------------KDGTGSVDATP
AN7572.1  	PEEAILPSIEDDLSPRNSPSPLALASRSALLTSDTKPPCSRYITKIHKRADSTASLESPK
          	  :             :.       . ..   . :....:   :.  .            

MG00345.1 	----SNGRPSGKSSQG------------------------------GQQPMNFI------
NCU07378.1	R-QLSLAKVDEEAAEA------------------------------DEEPTPRPLSRSVN
FG01312.1 	----RPPTVARDGCGE------------------------------DEEATPRPVGK---
AN7572.1  	RGQITPPVVFPSQSQNPSEAYPSTPPIVFDGPADSGPYQSSLTPIPDSDATPRPMHTPCP
          	 ..        . .  .:.: .::..   ...:.:.. .:: :. ...:.       .  

MG00345.1 	------------~-----------------------------------------------
NCU07378.1	Q-----------~-----------------------------------------------
FG01312.1 	------------~-----------------------------------------------
AN7572.1  	NPDTEKTPRPGR2LSTWHWFQSRKKPDQNLLQDCCIYMLASPMQCILTGTIYTVADAGKE
          	...:..:. ..   ::    .: ......  ....    ::. ..  :.:  : :.:...

MG00345.1 	--------------~
NCU07378.1	--------------~
FG01312.1 	--------------~
AN7572.1  	RCFPRLSARVRARE0
          	 . .  ::   : .
```
